# A comparative study of auto-contouring softwares in delineation of organs at risk in lung cancer and rectal cancer

**DOI:** 10.1038/s41598-021-02330-y

**Published:** 2021-11-26

**Authors:** Weijun Chen, Cheng Wang, Wenming Zhan, Yongshi Jia, Fangfang Ruan, Lingyun Qiu, Shuangyan Yang, Yucheng Li

**Affiliations:** 1 Department of Radiation Therapy, Zhejiang Provincial People’s Hospital, Affiliated People’s Hospital, Hangzhou Medical College, Hangzhou, 310014 Zhejiang People’s Republic of China; 2 Department of Nuclear Science and Technology, University of South China, Hengyang, 421001 Hunan People’s Republic of China; 3 Department of Radiation Therapy, Shanghai Pulmonary Hospital, Shanghai, 200433 People’s Republic of China

**Keywords:** Radiotherapy, Cancer imaging

## Abstract

Radiotherapy requires the target area and the organs at risk to be contoured on the CT image of the patient. During the process of organs-at-Risk (OAR) of the chest and abdomen, the doctor needs to contour at each CT image. The delineations of large and varied shapes are time-consuming and laborious. This study aims to evaluate the results of two automatic contouring softwares on OARs definition of CT images of lung cancer and rectal cancer patients. The CT images of 15 patients with rectal cancer and 15 patients with lung cancer were selected separately, and the organs at risk were manually contoured by experienced physicians as reference structures. And then the same datasets were automatically contoured based on AiContour (version 3.1.8.0, Manufactured by Linking MED, Beijing, China) and Raystation (version 4.7.5.4, Manufactured by Raysearch, Stockholm, Sweden) respectively. Deep learning auto-segmentations and Atlas were respectively performed with AiContour and Raystation. Overlap index (OI), Dice similarity index (DSC) and Volume difference (D_v_) were evaluated based on the auto-contours, and independent-sample *t*-test analysis is applied to the results. The results of deep learning auto-segmentations on OI and DSC were better than that of Atlas with statistical difference. There was no significant difference in D_v_ between the results of two software. With deep learning auto-segmentations, auto-contouring results of most organs in the chest and abdomen are good, and with slight modification, it can meet the clinical requirements for planning. With Atlas, auto-contouring results in most OAR is not as good as deep learning auto-segmentations, and only the auto-contouring results of some organs can be used clinically after modification.

## Introduction

In recent years, the incidence of thoracic and abdominal tumors is getting higher and higher, and most are Lung and Rectum tumors^1–3^. In order to improve the cure rate, about 70% of patients with malignant tumors need to receive radiation therapy. Radiotherapy has gradually become the second largest cancer treatment technology after surgery^4,5^. Radiotherapy requires the target area and the organs at risk to be contoured on the CT image of the patient. During the process of organs-at-Risk (OAR) of the chest and abdomen, the doctor needs to contour at each CT image. The delineations of large and varied shapes are time-consuming and laborious. Moreover, the OARs contours of the same patient by different doctors are subjectively different.

If automatic contouring of organs at risk is implemented, the efficiency of the doctors can be improved, and the subjective differences of contours by different doctors can be reduced. The efficiency and consistency of treatment are of great significance^6–9^. With the development of science and technology, the accuracy and efficiency of radiation therapy need to be improved. From automatic contour softwares available on the market, we have selected AiContour (version 3.1.8.0, Linking MED, Beijing, China) intelligent contouring system and Raystation (version 4.7.5.4, Research, Stockholm, Sweden) automatic delineating system to analyze the results of shape similarity compared to the contour from experience doctor. Index (Dice Similarity Coefficient, DSC), overlap index (OI) and volume difference (D_v_) were evaluated to analyze the accuracy of automatic contouring of thoracic and abdominal organs. Finally, independent sample t-test proofreading was performed with SPSS.

## Materials and methods

### General information

We select 15 patients with lung cancer and 15 patients with rectal cancer who were treated in Zhejiang Provincial People's Hospital from March 2019 to November 2019 randomly. The ethics institutional review board of Zhejiang provincial people’s Hospital approved the protocols for data collection and analyses. All patients imaging data applied for exemption from informed consent and approved by the ethics institutional review board of Zhejiang provincial people’s Hospital. All patients were placed in supine position during CT simulation, and a thermoplastic masks and vacuum cushions were selected based on clinical needs. The body position is fixed, and the scanning slice thickness is 5 mm. The patients’ CT were selected as target images, and the data template libraries of the two software were used for automatic sketching.

### Software tools

Using the Atlas Based segmentation function in Raystation and the artificial intelligence automatic cloud delineating software AiContour. Raystation uses an algorithm based on interactive information to select template from the Atlas library. Contour the best matching case of CT, deform the outline of the case, and map the result of the deformation to the CT to be delineated to form an automatically delineated outline^10^. The AiContour system is based on the segmentation network training of UNet^11^, forming recognition ability, so as to achieve the ability of automatic contour drawing. Except, The AiContour system available for any radiotherapy treatment panning system. The target CT images automatically contour by the two sets of software are from the scanning of the large-aperture four-dimensional CT simulator (version Discovery CT590, GE, Wisconsin USA).

### Contour content

For lung cancer patients, pancreas, spleen, stomach, liver, esophagus, heart, left lung, right lung, and spinal cord were selected as the contouring OARs. For rectal cancer patients, the OARs are left kidney, right kidney, spinal cord, left femoral head, right femoral head, left leg bone, right leg bone, pelvis, rectum, and bladder. The organs at risk manually contoured by the doctor on the Raystation planning system was selected as the standard structure, and the results of the automatic contours created by two auto-contouring software were respectively imported into the planning system and compared.

### Data analysis

Using the OAR outline manually contoured by the experienced doctor as a reference, the results from two software were assessed with the cross-reference OI, shape similarity index DSC, and volume difference D_v_ respectively.

1 $$ {\text{OI}} = \left( {{\text{V}}_{{\text{a}}} \cap {\text{V}}_{{\text{m}}} } \right)/{\text{V}}_{{\text{m}}} $$

2 $$ {\text{DSC}} = {2}\left( {{\text{V}}_{{\text{a}}} \cap {\text{V}}_{{\text{m}}} } \right)/\left( {{\text{V}}_{{\text{a}}} + {\text{V}}_{{\text{m}}} } \right) $$

3 $$ {\text{D}}_{{\text{v}}} = \left( {{\text{V}}_{{\text{a}}} - {\text{V}}_{{\text{m}}} } \right)/{\text{V}}_{{\text{m}}} $$

Among them, Va represents the volume (cm^3^) automatically contoured by the software, and V_m_ represents the volume (cm^3^) manually contoured by the doctor. Among them, the closer the OI index and the DSC index are to 1, and the closer the D_v_ value is to 0, the better the delineating result.

### Statistical methods

**Figure 1:**
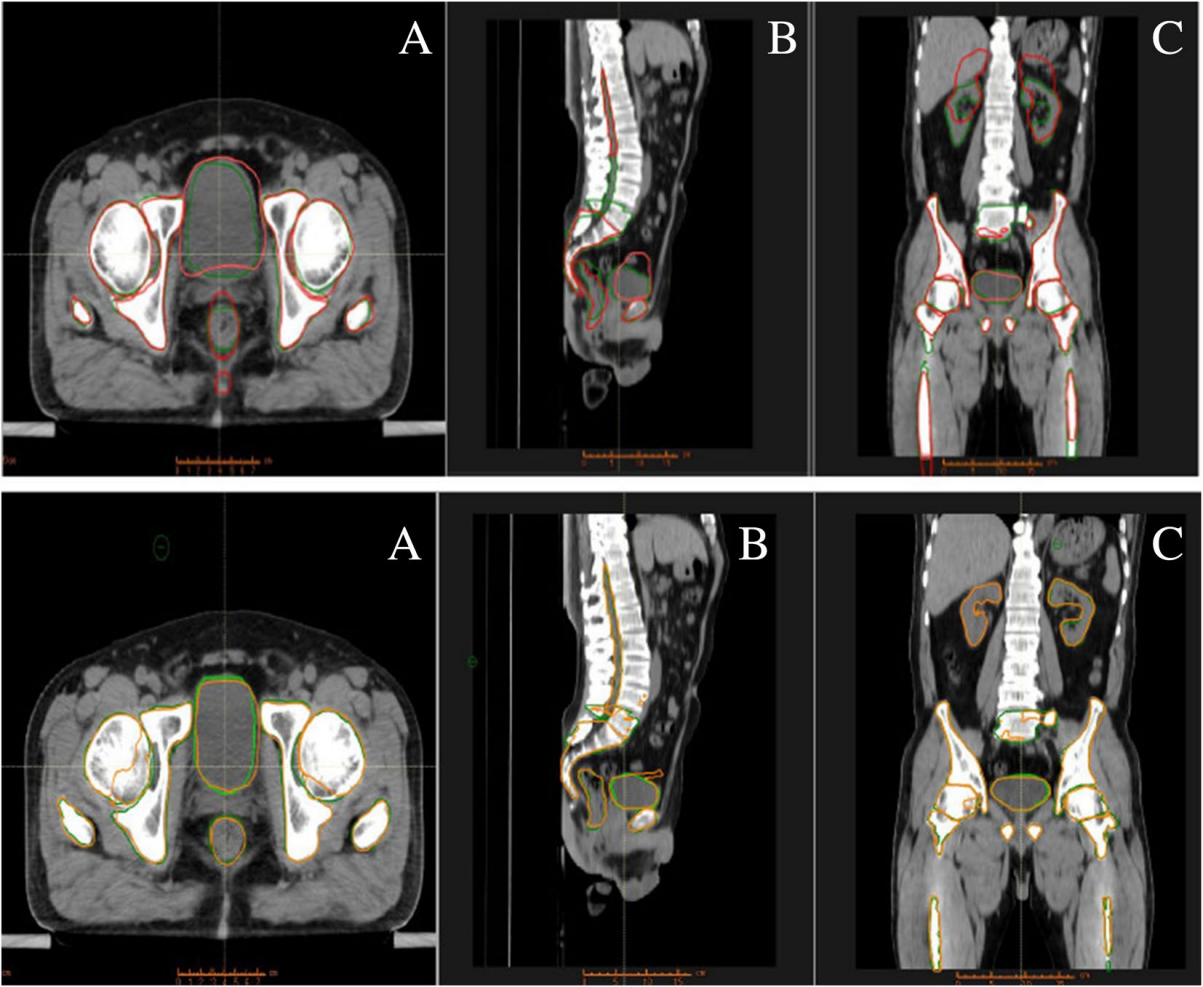
Comparison of OAR results of rectal cancer patients using two software. [(**A**) is the traverse plane, (**B**) is the sagittal plane, (**C**) is the coronal plane].

**Figure 2:**
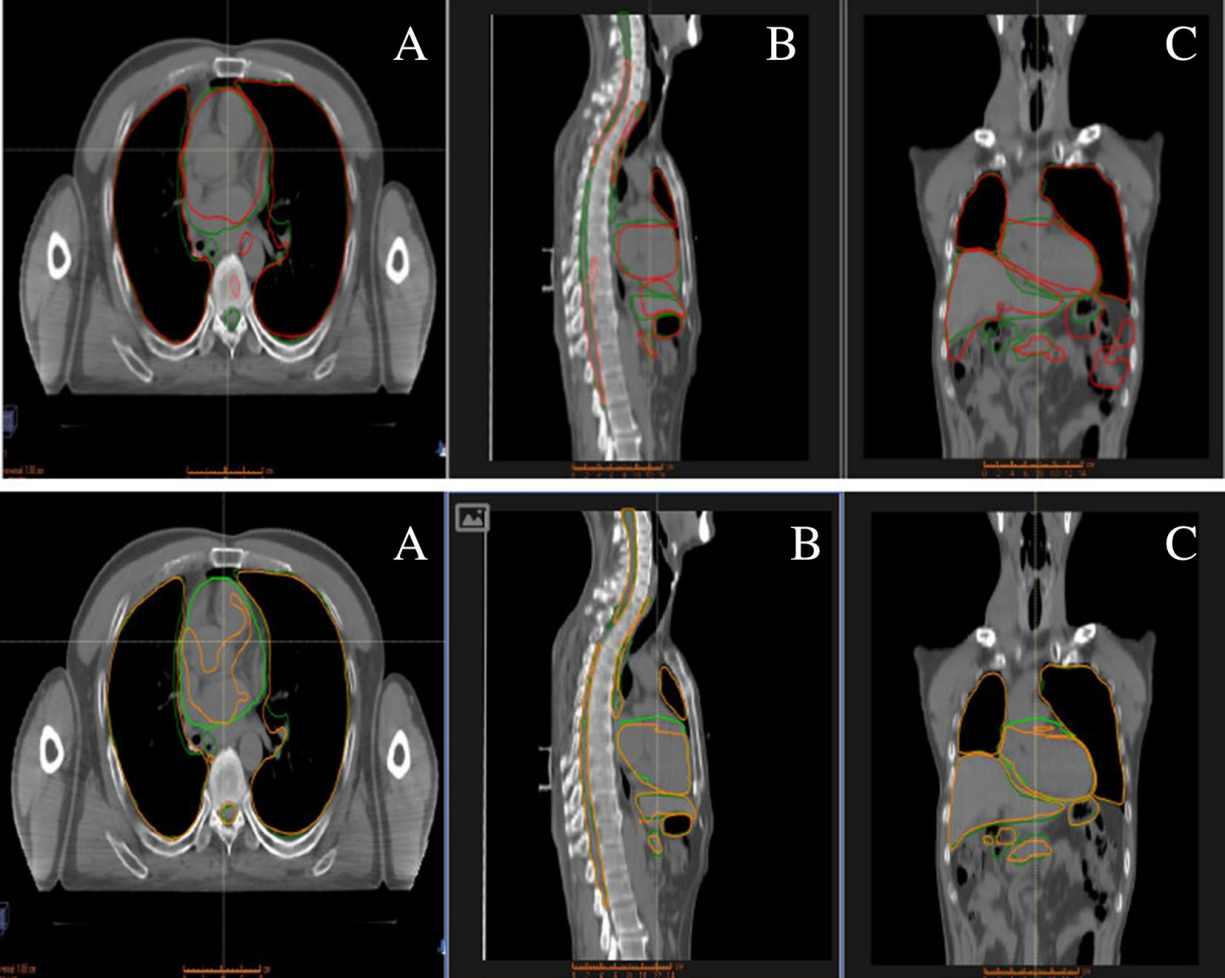
Comparison of OAR results of lung cancer patients using two software. [(**A**) is the traverse plane, (**B**) is the sagittal plane, (**C**) is the coronal plane].

SPSS 23.0 was used to perform independent sample *t*-test statistical analysis on the results contoured by the two software. The difference was statistically significant with *P* < 0.05, and the analysis was plotted by using Origin 8.0. (As shown in Figs. 1 and 2).

### Ethics approval and consent to participate

This study was approved by the ethics institutional review board of Zhejiang provincial people’s Hospital and conducted in accordance with the ethical standards of the Declaration of Helsinki.

## Results

We have analyzed OI, DSC, and D_v_ for patients with lung cancer and rectal cancer.

The results of OAR delineation with deep learning auto-segmentations in Lung cancer cases show that the average values of OI and DSC delineations of most organs are better than 0.8, and the mean D_v_ of most delineated organs are < 0.1. Among them, the right lung has the best OI, DSC and D_v_, and the mean OI is > 0.97. The average DSC is > 0.98, and the average D_v_ is − 0.003. The worst OI and DSC mean is the pancreas. The mean OI is less than 0.67, the mean DSC is less than 0.66, and the biggest difference between D_v_ is the esophagus, and the mean D_v_ is greater than 0.3. Compared with lung cancer, the OAR contour of rectal cancer is more stable. Both OI and DSC are greater than 0.8, and D_v_ are less than 0.1. In the spinal cord delineation of patients with lung and rectal cancer, rectal cancer delineation results are better in terms of various values.

Atlas results in lung cancer patients' OAR delineation show that the delineation results of different organs are quite diverse, and the delineation results of some organs are poor. Among DSC and OI, the best results of OI and DSC are left and right lungs. The mean OI and DSC values are both greater than 0.95. The best result of D_v_ is the right kidney. The mean value of D_v_ is 0.003. The lowest OI and DSC is the pancreas, with OI less than 0.32, DSC less than 0.28, and the worst D_v_ is the bladder, with an average D_v_ > 0.84. The interval of some data of Atlas is relatively large. It may be that in some patients, the library does not have a good CT case with the CT to be outlined, and the deformation registration ability is insufficient, resulting large differences in the results. The comparison results of the two softwares are shown in Tables 1 and 2.

**Table 1:** Comparison of OI and DSC for OAR automatic delineation of rectal cancer by two software.

	Left kidney	Right kidney	Spinal	Left femur head	Right femur head	Left leg bone	Right leg bone	Pelvis	Rectum	Bladder
**OI**										
AiContour	0.971	0.964	0.972	0.936	0.945	0.963	0.969	0.942	0.870	0.836
Raystation	0.492	0.631	0.715	0.896	0.904	0.891	0.884	0.881	0.528	0.592
*t*	7.630	5.929	4.465	1.867	1.810	3.391	3.743	3.617	4.779	2.284
*P*	0	0	0.001	0.072	0.081	0.003	0.002	0.002	0	0.030
**DSC**										
AiContour	0.952	0.956	0.943	0.926	0.937	0.975	0.974	0.957	0.874	0.805
Raystation	0.492	0.632	0.687	0.878	0.883	0.877	0.870	0.895	0.439	0.448
*t*	8.198	6.042	5.156	3.808	3.262	5.777	5.762	5.907	7.599	4.057
*P*	0	0	0	0.001	0.003	0	0	0	0	0
**D**_**v**_										
AiContour	0.039	0.017	0.061	0.022	0.017	− 0.026	− 0.011	− 0.031	− 0.012	0.081
Raystation	− 0.015	0.003	0.056	0.041	0.049	0.036	0.037	− 0.033	0.390	0.843
*t*	0.577	0.217	0.051	0.554	− 1.081	− 1.284	0.020	0.083	− 2.583	− 1.473
*P*	0.569	0.831	0.960	0.584	0.289	0.219	0.984	0.935	0.021	0.163

**Table 2:** Comparison of OI and DSC for OAR automatic delineation of lung cancer by two software.

	Pancreas	Spleen	Stomach	Liver	Esophagus	Heart	L Lung	R Lung	Spinal
**OI**									
AiContour	0.664	0.916	0.836	0.972	0.860	0.916	0.973	0.979	0.944
Raystation	0.315	0.670	0.459	0.886	0.573	0.816	0.959	0.955	0.671
*t*	6.094	4.121	7.167	5.414	4.558	2.766	1.972	4.155	7.972
*P*	0	0.001	0	0	0	0.010	0.059	0	0
**DSC**									
AiContour	0.650	0.920	0.832	0.965	0.751	0.925	0.977	0.981	0.896
Raystation	0.277	0.492	0.471	0.800	0.398	0.858	0.956	0.960	0.583
*t*	7.139	8.804	6.899	14.214	10.194	2.486	3.476	4.345	9.565
*P*	0	0	0	0	0	0.019	0.002	0	0
**D**_**v**_									
AiContour	0.066	− 0.010	0.010	0.015	0.301	− 0.019	− 0.010	− 0.003	0.107
Raystation	0.544	0.824	− 0.033	0.222	0.832	− 0.109	0.007	− 0.010	0.355
*t*	− 1.292	− 2.794	0.514	− 4.029	− 2.278	2.224	− 1.078	1.309	− 2.179
*P*	0.214	0.014	0.614	0.001	0.037	0.034	0.290	0.204	0.046

## Discussions

This paper was a comparative contouring between two deep learning auto-segmentations and Atlas. The results of this study that contouring of artificial intelligence is better than Atlas. The deep learning auto-segmentations is more similar to clinicians manual sketch’s OARs, and greatly saves the physician's working time.

In the design of radiation treatment plans, the accuracy of organ at risk contour often affects the dose distribution in patients and affects the actual target area and the dose of organ at risk, then impacts the treatment quality ultimately^12,13^. Under the development of science and technology, the automatic delineating performance has also been continuously optimized and improved. Due to the high precision of the automatic delineating technology, doctors can use it for clinical purpose with only slight modifications, reducing unnecessary workload for clinicians. It also greatly improves the treatment efficiency.

During the manual delineation of the organs in the chest and abdomen, the time of manual delineation of one patient’s OAR is about 1.5 h. deep learning auto-segmentations is usually about 40 s while Atlas takes about 5 min. These are consistent with the results of Lustberg T et al.’s studies^14^, the median time of manual contouring is 20 min, that the total median time saved is 7.8 min when using atlas-based contouring and 10 min for deep learning contouring. The delineating time is different due to different software and contouring organs. Deep learning auto-segmentations greatly saves time, because deep learning auto-segmentations is a model established by artifical intelligence. Due to its own database of convolution neural network learning, a good contour of organ at risk is achieved^15^. In terms of Atlas, the target image and database image deformation registration technology is implemented clinically, that the algorithm is not excellent enough, leading the result of the contour is not always satisfied.

**Figure 3:**
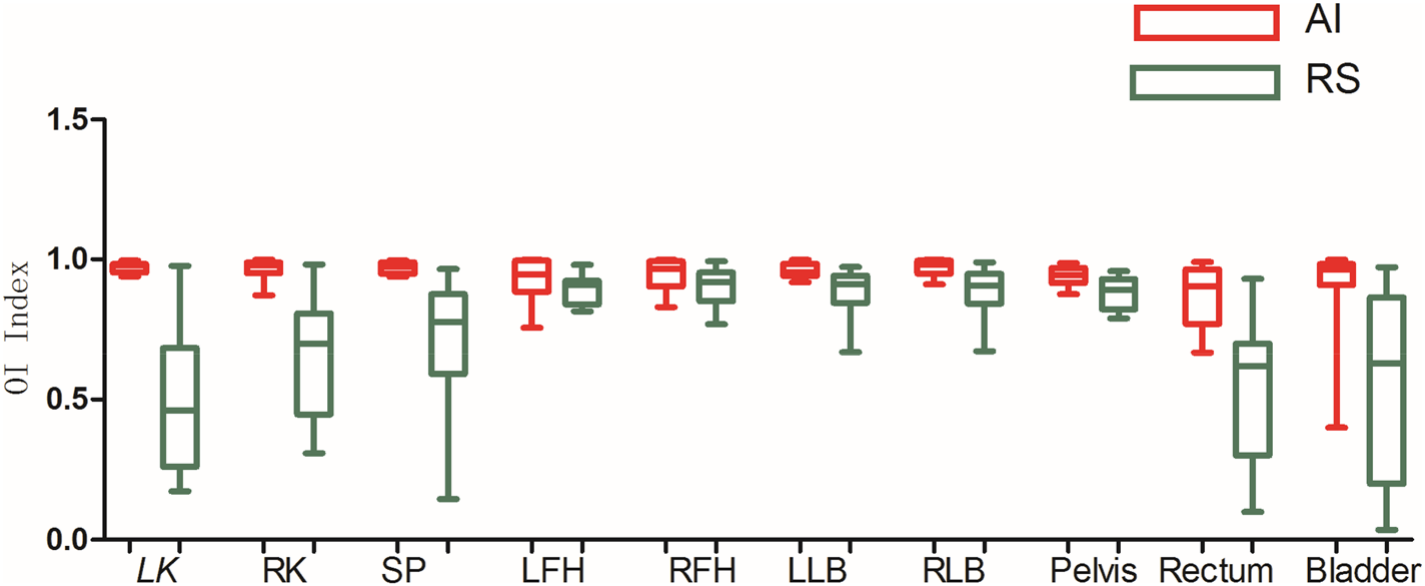
Comparison results of OI between two software in automatic delineation of rectal cancer OAR (Notation: LK means left kidney, RK means right kidney, SP means spinal cord, LFH means left femoral head, RFH means right femoral head, LLB means left leg bone, RLB means right leg bone).

**Figure 4:**
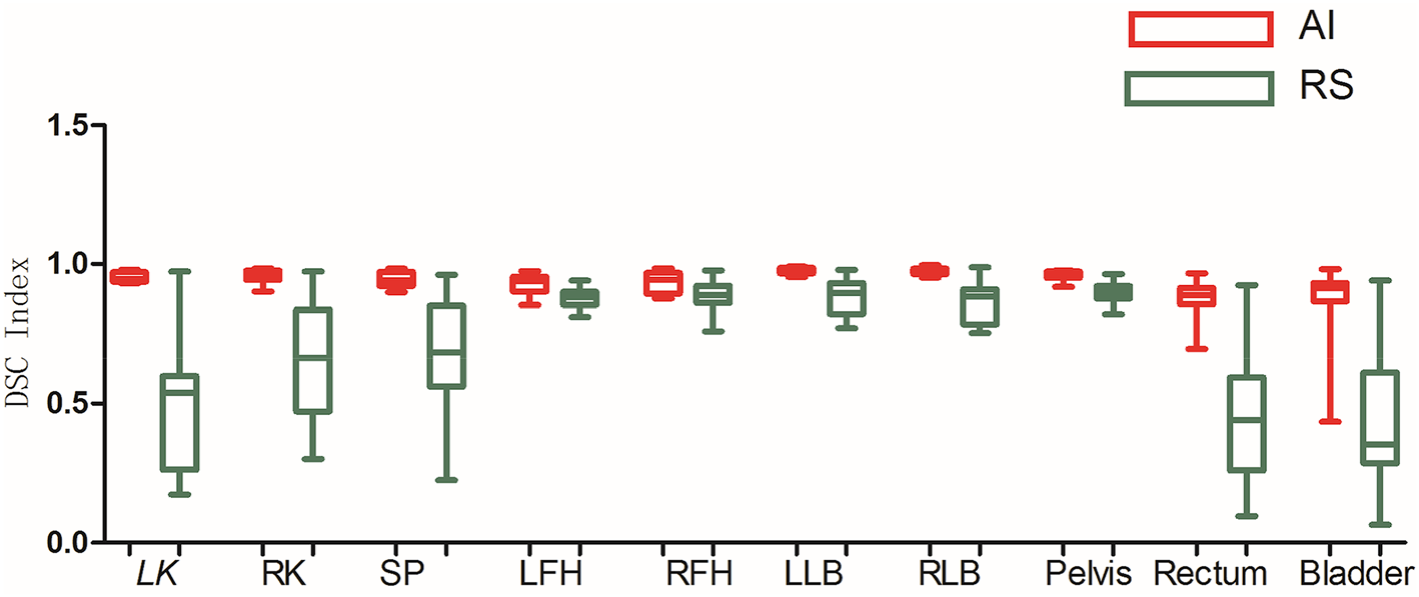
Comparison results of DSC between two software in automatic delineation of rectal cancer OAR.

**Figure 5:**
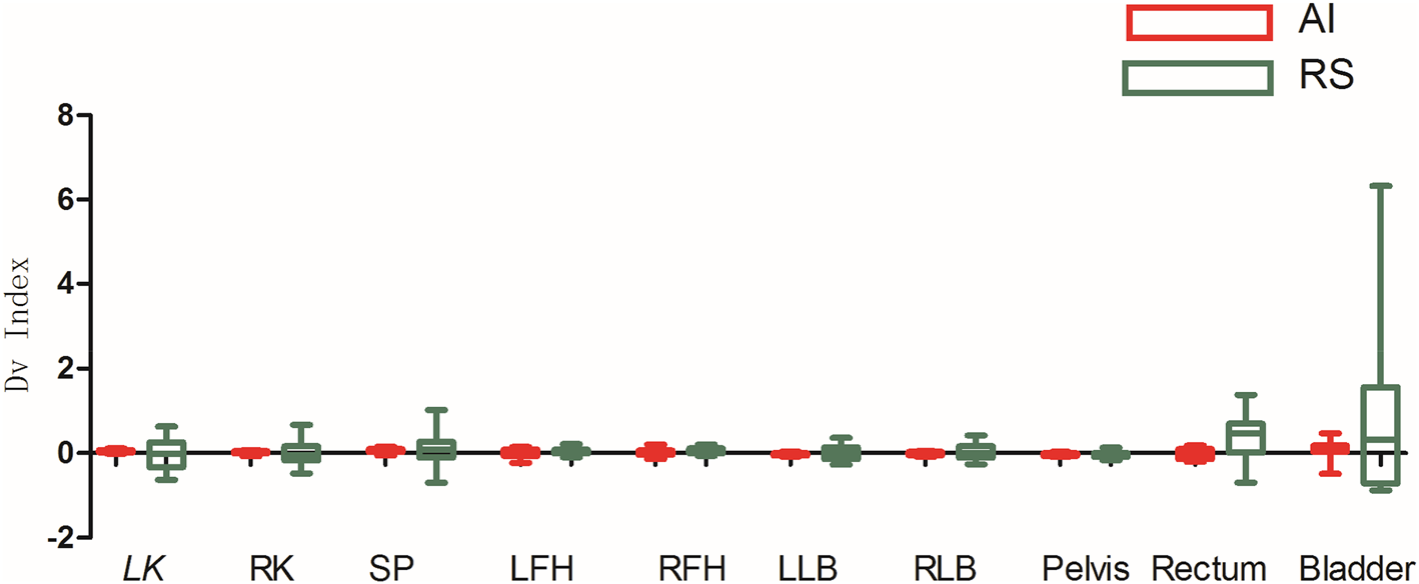
Comparison results of D_v_ between two software in automatic delineation of rectal cancer OAR.

**Figure 6:**
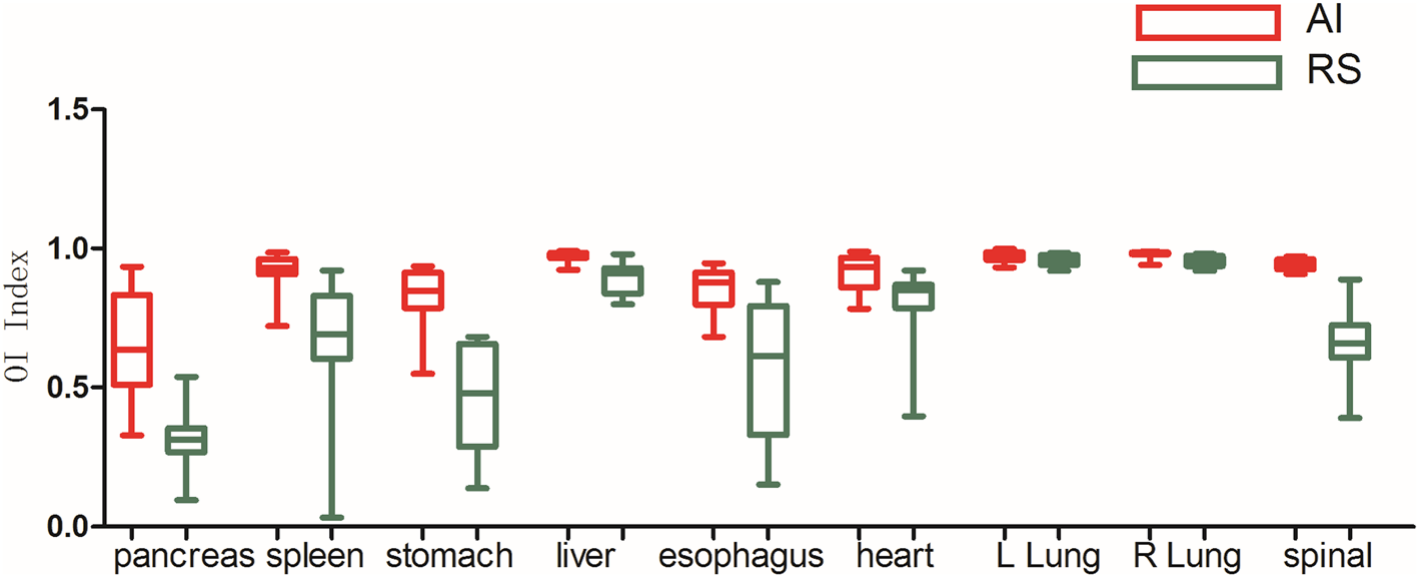
Comparison results of OI between two software in automatic delineation of lung cancer OAR.

**Figure 7:**
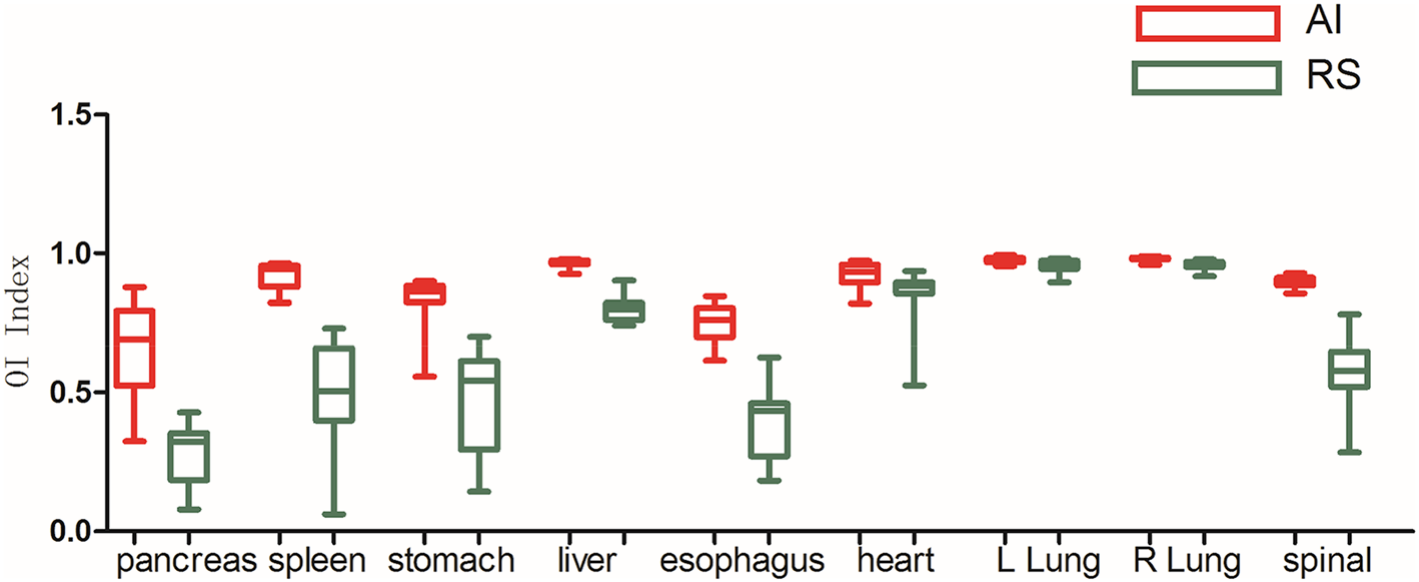
Comparison results of DSC between two software in automatic delineation of lung cancer OAR.

**Figure 8:**
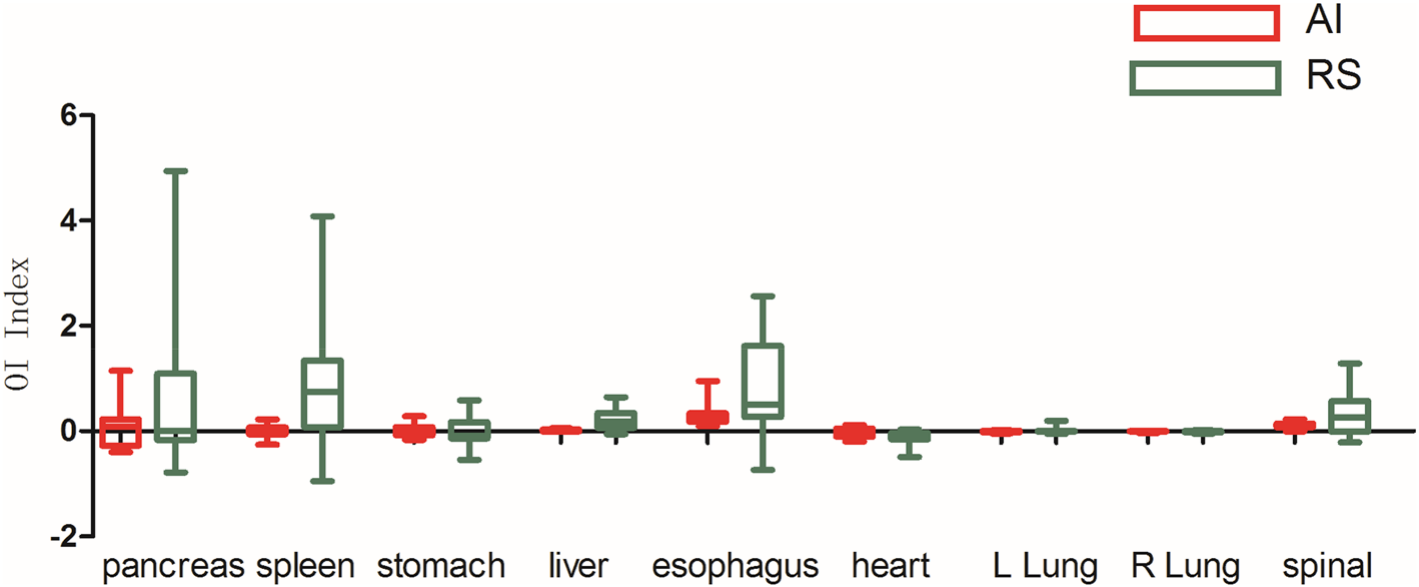
Comparison results of D_v_ between two software in automatic delineation of lung cancer OAR.

As shown in Figs. 3, 4, 5, 6, 7, and 8, deep learning auto-segmentations has a relatively concentrated distribution value in the data distribution, and the density of the upper and lower bounds are not much different. When there is obvious difference in density in the automatic delineating process, the results have a good match with the "gold standard" manually delineated by the doctors^16–19^. Due to the obvious difference between the density of leg bones and lungs and the density of surrounding tissues, there is not much difference between Atlas and deep learning auto-segmentations in the delineation of the leg bones and the bilateral lungs. Both areas have reached a comparative level (DSC > 0.7) because of the contrast between bones and soft tissue, which can be used clinically with only partial modification^20^. However, limited by the differences of eating, drinking, digesting and physical health among peoples, the target contouring for digestive organs, such as rectum, bladder, stomach, pancreas et al., become more hard. The worst results from the two softwares were showing in the pancreas. The low contrast in the boundary between the pancreas and the surrounding tissue under ordinary CT scans may cause the boundary not to be defined clearly, resulting in poor results. The D_v_ value of most of the contour results may not be statistically significant due to the difference between the positive and negative data^21^.

The spinal cord is contoured in CT images of lung cancer and rectal cancer, and independent samples are tested. The two results are statistically different (*P* < 0.05). The difference between the two groups of data is probably due to the small volume of the spinal cord itself. Individual and statistical differences caused by the data of the contour results are more significant. According to the OI and DSC of results from deep learning auto-segmentations, the delineation results of different positions of the spinal cord is relatively close. Although the delineation results are different, the data distribution remains relatively centralized, which shows that deep learning auto-segmentations delineating results are stable. From the theory of auto-contouring, Atlas needs to match the best case in the database for deformation registration. The U-net network is a CNN (Convolutional Neural Network) -based image segmentation network. It uses a computer to input two-dimensional or three-dimensional images to the image pixels. Each image pixel is assigned a weight coefficient and weight quality. By translation and frequency conversion, it is trained to find a feature template suitable for classification, and can quickly filter and match feature pixels on the grid of the image during reading^22–25^. Atlas, due to the inconsistency of organs as different ages and shapes, it is difficult to establish a universal map. The registration process is deformable, which is time consuming. The accuracy of deep learning auto-segmentations is based on the raw data of delineating accuracy results, and the output accuracy is not necessarily the optimal solution of the delineating results, and optimization learning is required. Compared with the contouring method of Atlas, deep learning auto-segmentations is more stable in the speed and accuracy of the chest and abdomen organs and has certain advantages.

## Conclusions

Both methods can be used for automatic contouring. Deep learning auto-segmentations achieves better contouring results on OARs delineate of lung cancer patients and rectal cancer patients. Atlas the delineation is good for lung and heart, and the result for the femoral head is good in patients with rectal cancer. However, deep learning auto-segmentations is better in both scenario and can be used clinically. Both software are not ideal for the contour of the pancreas, so the algorithm of image segmentation needs to be optimized on the less obvious parts of tissues and organs to have two software used in the automatic delineation of chest and abdomen organs. Limited by the differences of eating, drinking, digesting and physical health among peoples, the OARs become more hard. Clinicians should be reviewed and confirmed OARs before it be used in clinical practice. Finally, this study did not add the evaluation of parameters such as normalized surface dice, Hausdorff distance, etc. The author will evaluate these parameters in subsequent studies.

## Data Availability

The data are not available for public access because of patient privacy concerns, but are available from the corresponding author on reasonable request.
