# Author Correction: A comparative study of auto-contouring softwares in delineation of organs at risk in lung cancer and rectal cancer

**DOI:** 10.1038/s41598-024-54103-y

**Published:** 2024-02-12

**Authors:** Weijun Chen, Cheng Wang, Wenming Zhan, Yongshi Jia, Fangfang Ruan, Lingyun Qiu, Shuangyan Yang, Yucheng Li

**Affiliations:** 1 Department of Radiation Therapy, Zhejiang Provincial People’s Hospital, Affiliated People’s Hospital, Hangzhou Medical College, Hangzhou, 310014 Zhejiang People’s Republic of China; 2 Department of Nuclear Science and Technology, University of South China, Hengyang, 421001 Hunan People’s Republic of China; 3 Department of Radiation Therapy, Shanghai Pulmonary Hospital, Shanghai, 200433 People’s Republic of China

Correction to: *Scientific Reports* 10.1038/s41598-021-02330-y, published online 26 November 2021

The original version of this Article contained an error in the grant number in the Acknowledgements section.

“This research was partially supported by the Zhejiang Basic Public Welfare Research Project (GF21H180053) and the Zhejiang Medical and Health Science and Technology Plan Project (2021PY002).”

now reads:

“This research was supported by Zhejiang Province Natural Science Foundation of China under Grant No. LGF21H180014 and the Medical and Health Research Project of Zhejiang Province under Grant No. 2021PY002.”

The original Article has been corrected.

